# In Situ Calcium Carbonate Mineralization of Mycelium Composites: Processing Challenges and Physical-Mechanical Property Implications

**DOI:** 10.1021/acsomega.5c13225

**Published:** 2026-03-02

**Authors:** Stefania Akromah, Neha Chandarana, Stephen J. Eichhorn

**Affiliations:** † Bristol Composites Institute, School of Civil, Aerospace and Design Engineering, 1980 University of Bristol, University Walk, Bristol BS8 1TR, U.K.; ‡ School of Chemistry, University of Bristol, Cantock’s Close, Bristol BS8 1TS, U.K.

## Abstract

The potential for calcium carbonate (CaCO_3_) mineralization of mycelium composites (MCs) using a procedure commonly applied to natural wood is explored. The effects of this mineralization on the structural, thermal, and compressive properties of the materials are explored, revealing unexpected outcomes that challenge prior observations reported for mineralized wood. CaCO_3_ was deposited into MCs via an in situ, vacuum-assisted mineralization process using calcium acetate and sodium bicarbonate, with treatment durations of 5 and 24 h, resulting in mineral contents of ∼1 and 10 wt %, respectively. Fourier transform infrared spectroscopy (FTIR), scanning electron microscopy (SEM), and selective area electron diffraction (SAED) confirmed the formation of CaCO_3_ crystals, identifying the predominant polymorphs; namely vaterite and calcite. Notably, mineralization led to a reduction in the thermal stability of the composites, suggesting a potential catalytic effect of CaCO_3_ on the thermal degradation of the MC. Compressive testing of the MCs further indicated a decline in their mechanical strength following mineralization, likely attributable to structural alterations induced during the process. In addition, Water Contact Analysis (WCA) showed a substantial decrease in surface hydrophobicity following mineralization, with contact angles reduced by more than 50% relative to untreated MCs. This study underscores the need for careful evaluation of mineralization strategies, as they may lead to deterioration rather than enhancement of MC performance. This is found to be contrary to many other previous reports on the mineralization of biomass materials.

## Graphical abstract

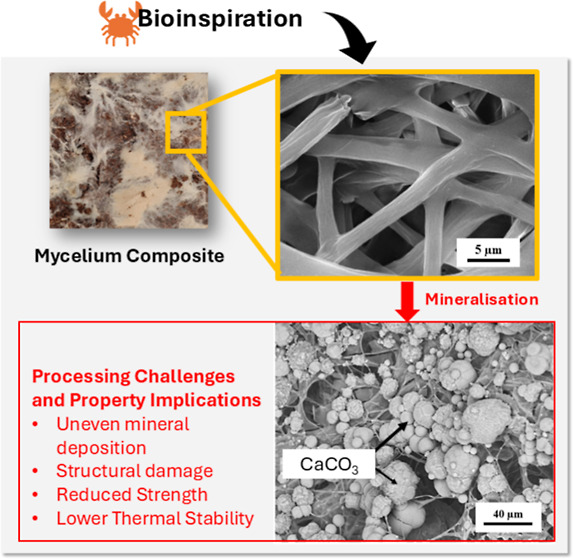

## 1 Introduction

Mycelium composites (MCs) are usually made of fungal mycelium and lignocellulosic substrates, where mycelial hyphae bind the substrate particles.^1^ MCs are considered a sustainable alternative to oil-based polymers, and are commonly used in nonload-bearing applications such as packaging,^2^ insulation, and horticultural^3^ products, where their physical properties are particularly advantageous. However, their poor mechanical performance poses a significant limitation to their broader application.^4^ The relatively weak interfacial interactions between the mycelium matrix and lignocellulosic particles, primarily governed by physical entanglements and weak secondary bonds, result in a tensile strength that can be as low as 0.01 MPa for MCs based on Pleurotus fungi.^1,5^ Additionally, they have low densities (typically between 0.10 and 0.40 g cm^–3^),^6,7^ arising from the inherent porosity of both the mycelial network and the lignocellulosic substrates used.^1^ This low density contributes to reduced compressive strengths for the materials (typically <2 MPa for as-prepared unmodified MCs).^8^

The mycelium itself consists of intertwined filaments (hyphae) that form a complex, fibrous network with an open-cell structure. Moreover, mycelial hyphae contain a high level of nonstructural elements such as proteins, lipids, and amorphous glucans, which makes them mechanically weak.^1,6,9^ Lignocellulosic biomass is also intrinsically porous, and particle characteristics such as shape, size, and distribution can influence the bulk density of the composite in multiple ways. For example, while fine particles such as saw dust increase the density contribution of the reinforcement phase (i.e., the substrate), they can also limit aeration for fungal growth through the core during manufacturing, thereby reducing mycelium density and contact surfaces.^1,6^

Various approaches have been explored to increase the density of MCs to enhance their compressive performance. Key strategies involve optimizing fungal species selection,^10^ substrate formulations,^11^ and process conditions (e.g., substrate composition, pH, moisture content, and oxygen diffusion),^12,13^ to promote mycelial proliferation and obtain denser and more cohesive matrices. For example, simultaneous decay fungi such as *Trametes hirsuta* and *Trametes versicolor* are capable of degrading multiple complex polysaccharides within a single system, enabling faster growth and denser mycelial development.^14^ Nonetheless, even in such cases, nutrient-rich substrates (e.g., those high in sucrose or starch) are still recommended, as they are more easily digested by fungi.^15^ In contrast, lignocellulosic biomass has a more complex and stable structure, requiring fungi to secrete large amounts of enzymes to break it down into assimilable nutrients, which ultimately results in lower bioconversion yields.^16^ In practice, the selection of biomass is largely constrained by accessibility, cost, and the need to prioritize nutrient-rich crops for human and animal consumption, thereby making lignocellulosic biomass the preferred choice for MC production.^17^ Hence, there is a need for alternative methods that can effectively improve the strength of MCs, regardless of the challenges arising from the use of these substrates.

Hot-pressing is the primary method of improving densification in MCs, and this approach has been shown to increase elastic modulus, stiffness, and flexural strength among other mechanical properties.^6^ These improvements are attributed to several factors: enhanced bulk density and reduced porosity; heat-induced polymerization of lignin and esterification reactions;^18,19^ and heat-induced cross-linking between amino acids in the mycelium cell walls and functional groups in the biomass.^19^ Another popular strategy for increasing densification involves the incorporation of organic and/or inorganic particles in MCs. For instance, Elsacker et al.^20^ reported that the incorporation of bacterial cellulose via coculture with *T. versicolor* coupled with heat pressing at 200 °C resulted in MCs with a high flexural strength (∼3 MPa) and flexural modulus (reported as 0.44 GPa). This was attributed to both enhanced heat-induced internal bonding and structural reinforcement from the bacterial cellulose–mycelium network.^20^ In another study, the addition of 2.5% cellulose nanofibrils (CNFs) led to a substantial increase in mechanical performance, with Young’s modulus increasing by up to 5-fold and ultimate strength by up to 7-fold, along with notable gains in the modulus of rupture.^21^ On the other hand, nanoclay particles were reported to increase compressive stiffness in MCs from ∼0.3 MPa to ∼0.5 MPa, although these effects are not consistently observed and often depend on nanoclay dispersion and fungal compatibility.^22^

Although processing strategies such as hot-pressing and the incorporation of nanofillers have shown promise, these approaches represent only one route to reinforcing mycelium composites. An alternative, bioinspired pathway is to exploit biomineralization processes to introduce inorganic phases within the MC structure. The present work represents the first direct application of a wood-derived calcium carbonate (CaCO_3_) mineralization protocol to MCs, implemented via in situ solution exchange. In the context of this article, mineralization refers to the precipitation and deposition of CaCO_3_ within and around an MC scaffold, a process that attempts to mimic the reinforcement mechanisms of some of nature’s strongest structural materials, such as nacre and bone. The method employed in this work follows the approach described by Choi et al.^23^ in which CaCO_3_ was successfully precipitated within the structure of natural (unmodified) wood, as confirmed by scanning electron microscopy (SEM) and X-ray computed tomography (CT). Similarly, Merk et al.^24^ successfully deposited vaterite and calcite within Norway spruce and European beech wood which resulted in an enhanced fire resistance.

Interestingly, although both Choi et al.^23^ and Merk et al.^24^ employed untreated wood and multiple solution infiltration cycles, their CaCO_3_ deposition outcomes differed significantly. Merk et al.^24^ reported dense and uniformly distributed CaCO_3_ with a loading of 20–35% in European beech wood. In contrast, Choi et al.^23^ observed a much lower volume fraction of CaCO_3_, which was sparsely distributed and poorly bonded within the wood, resulting in no significant mechanical increase. This discrepancy may be due to differences in the compositional and structural characteristics of the wood species used, which Choi et al. did not specify, making direct comparison difficult. Notably, another study reported that CaCO_3_ mineralization caused no significant change in the mechanical strength of tropical hardwoods (including *Cedrela odorata*, *Cordia alliodora*, and *Enterolobium cyclocarpum* species).^25^ In contrast, CaCO_3_-mineralized *Paulownia* wood, another hardwood type, exhibited a substantial increase in its compressive strength (from ∼22 MPa to ∼32 MPa) and Young’s modulus by ∼53%.^26^

Chemical and physical modifications of the organic scaffolds used for mineralization have also been employed to improve deposition yields. For example, predelignification of balsa wood, which exposes more hydroxyl functional groups for nucleation, has been shown to increase compressive strength from 3.15 MPa (native balsa) to 3.66 MPa (mineralized balsa).^27^ Similarly, introducing sodium alginate as a nucleation site to NaOH-pretreated poplar wood showed promising results, with a 16% increase in compressive strength and a 38% increase in flexural strength.^28^ The wooden artificial nacres (WANs) developed by Qiu et al.^29^ exhibited exceptional mechanical performance, including a bending strength of 93.3 MPa, a toughness of 7.4 MPa m^0.5^, a tensile strength of 122.6 MPa, and a work of fracture of 4.6 MJ m^3^. The specific strength significantly surpassed that of both natural nacre and other artificial mimics, despite a low density of just 159 kg m^–3^. These WANs were made by delignifying natural balsa wood and modifying it with maleic anhydride to introduce anionic carboxyl groups for Ca^2+^ bonding. The resulting material exhibited a multiscale architecture characterized by lamellar layering, organic bridging, and microroughness.

The growing body of research on biomineralization consistently highlights three key requirements: (1) a structural scaffold to support mineral growth; (2) an organic matrix, e.g., acidic proteins, collagen, or polysaccharides, with functional groups serving as nucleation sites that promote controlled calcium binding and deposition; and (3) a supply of calcium (Ca^2+^) and carbonate ions (CO_3_^2–^) to induce mineral deposition.^30,31^ The porous structure of MCs, along with the protein-rich composition of the mycelial cell wall, provides both a suitable scaffold and carboxylate (−COO^–^) nucleation sites. Additionally, partial delignification of the lignocellulosic substrate—resulting from the ligninolytic activity that white-rot fungi such as *P. eryngii* use to access cellulose and hemicellulose—may expose additional hydroxy (−OH) groups,^32–36^ offering further potential sites for mineral nucleation. As such, no chemical pretreatment was applied in this work, with biologically induced delignification considered sufficient. Calcium acetate (Ca­(CH_3_COO)_2_) and sodium bicarbonate (NaHCO_3_) were selected as the calcium and carbonate ion precursors, respectively. This study investigates the in situ mineralization of as-prepared MCs using CaCO_3_, with the aim of evaluating its impact on physical and compressive properties. It also identifies key limitations of the process and offers preliminary insights to guide the future development of mineralized biocomposites.

## 2 Materials and Methods

### 2.1 Materials

Coconut coir bricks (650 g per unit brick) were purchased from Divchi (Bradford, UK). *P. eryngii* (king oyster) spawns were obtained from Prodac Ltd. (London, UK). Gypsum (calcium sulfate dihydrate 99%) was purchased from APC Pure (Manchester, UK) and added to provide sulfur and calcium to support fungal growth; it also serves as a pH buffer (maintaining a near-neutral pH), preventing substrate acidification during mycelium growth which would otherwise promote bacterial contamination.^37–39^ Calcium acetate (99%, APC Pure, Manchester, UK) was used as the calcium ion (Ca^2+^) precursor for calcium carbonate mineral formation. Sodium hydrogen carbonate (sodium bicarbonate, Sigma-Aldrich, Gillingham, UK) served as the carbonate ion (CO_3_^2–^) precursor.

### 2.2 MC Manufacturing

The MC manufacturing methodology used in the present study is summarized in Figure 1. Coconut coir bricks were sterilized by pasteurization, following a similar procedure as used for mushroom substrates.^40,41^ For each coir brick (650 g), 50 g of gypsum was added to 4.5 L of water and boiled for 30 min. The boiling mixture was poured onto the coir bricks in a sterilized container and left for 5 min to allow them to absorb the water and expand, after which the hydrated coir was stirred for homogenization. The container was then covered, wrapped in polyester felt fabric (breather cloth), and left undisturbed for 24 h to allow sufficient time for substrate hydration and microbial sterilization. The breather cloth helped to reduce heat loss, ensuring there would be enough time for sterilization to occur. After 24 h, the sterile substrate was inoculated with mycelium spawn and rehomogenized to ensure even fungal distribution. The container was then covered, wrapped in breather cloth, and left undisturbed in a clean, dark space with the temperature set to 23 ± 2 °C.

**Figure 1:**
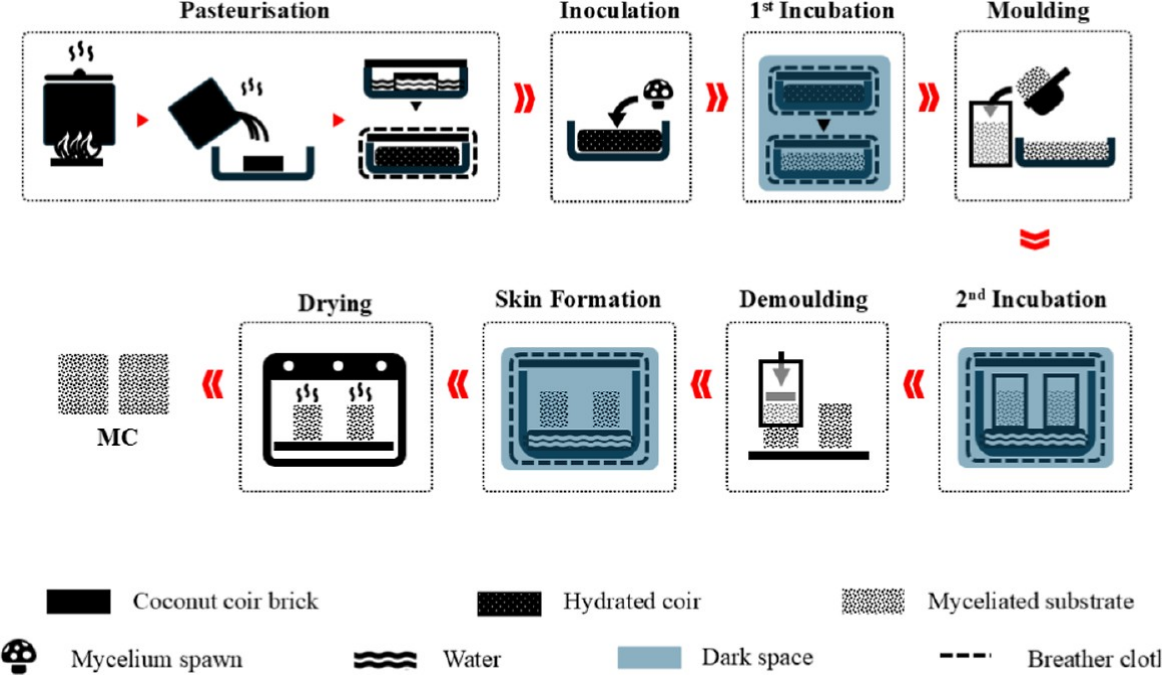
Schematic of the MC manufacturing method summarizing the key manufacturing steps: pasteurization and hydration of the coconut coir; inoculation with *P. eryngii* grain spawn; first incubation; molding of the myceliated substrate; incubation of the molded myceliated substrate (second incubation); removal of the as-grown MC from molds; placement of as-grown MCs in a saturated humid chamber for mycelial skin formation; drying of MCs for 48 h at 90 °C.

MCs were manufactured using the double-incubation method.^42^ In the first stage, inoculated substrates were incubated for 2 weeks to allow for mycelium proliferation, providing a higher number of growth points for efficient mycelial expansion in the second incubation stage. After the first incubation, the myceliated substrates (defined as substrates on which mycelium has grown) were homogenized for a uniform distribution and poured into 80 × 80 mm clear acrylic molds. The molds were filled with enough material to form 80 × 80 × 80 mm cubes, corresponding to approximately 375 g of myceliated substrate. The filled molds were positioned above a sterilized water bath inside a sealed chamber, where the rising water vapor created a near-saturated humidity environment (>95% RH) that prevented substrate drying and supported consistent mycelial growth. The setup was covered, wrapped again, and stored in a sterilized dark space. After 4 weeks, the samples were pressed, extruded manually from the molds, and placed back in the water bath for 48 h to allow the formation of a mycelium skin. Finally, the samples were dried in an oven at 90 °C for 48 h.

### 2.3 Mineralization Procedure

The MCs were mineralized using the method described by Choi et al.^23^ CaCO_3_ was selected to maintain direct comparability with this protocol, thereby enabling an unambiguous assessment of its transferability to mycelium composites. A combination of vacuum, gravity, and mechanical assistance was employed to enhance the infiltration of calcium and carbonate ions into the composites. Figure 2 presents a schematic flowchart of the mineralization process. After weighing and measuring the mycelium composite samples, they were placed in a sealed tub containing a 0.2 M calcium acetate solution. A vacuum was applied in short cycles (20 s each), with 1 min intervals between to allow gradual pressure adjustment and minimize sample damage. Vacuum was applied using a VW VP 86 pump at an absolute pressure of 100 mbar (∼1.45 psi). Once the air bubble release (outgassing) had significantly slowed, the vacuum was released, and the composites were left undisturbed in the calcium acetate solution for 2 h.

**Figure 2:**
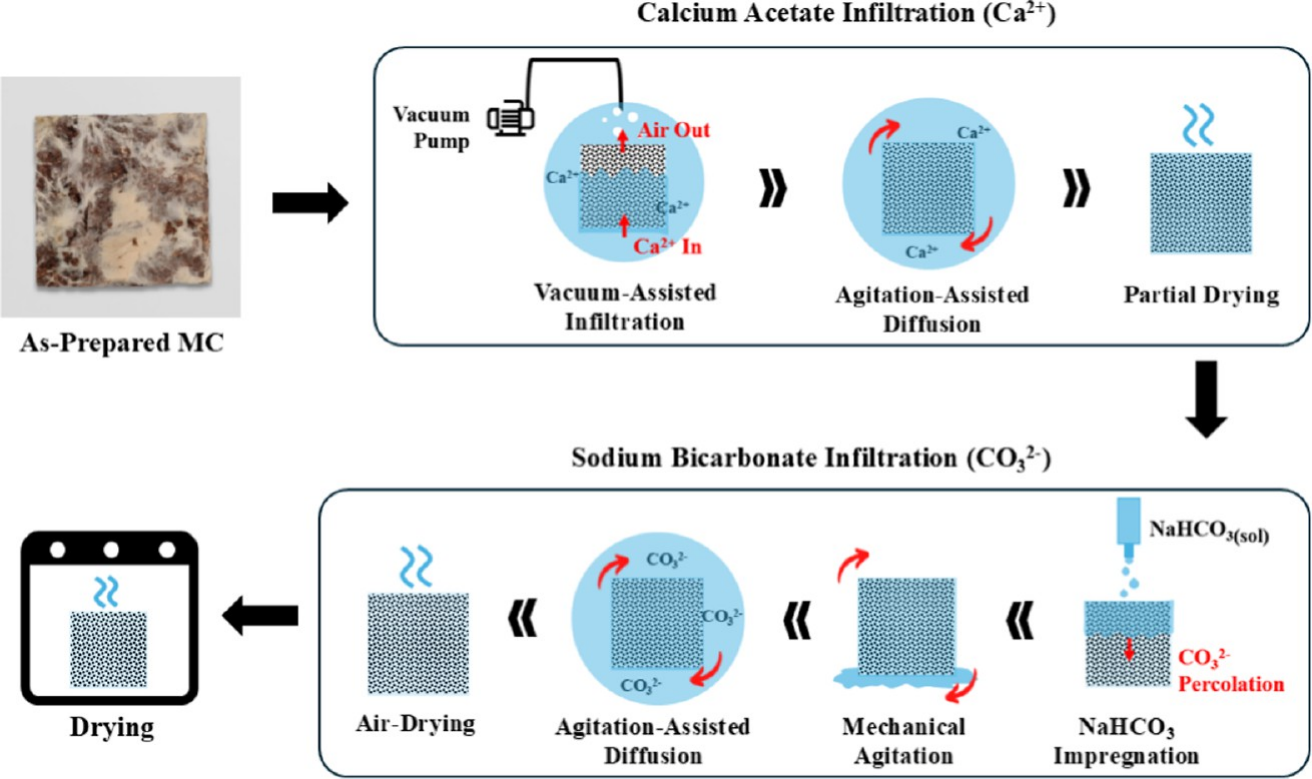
Flow diagram of the mineralization method, involving vacuum-assisted infiltration and agitation-assisted diffusion of calcium ions (Ca^2+^); gravity-assisted percolation and agitation-assisted diffusion of carbonate ions (CO_3_^2–^); and drying.

The number of vacuum cycles required was sample-dependent due to the intrinsic variability of as-prepared MCs, a common challenge when using these approaches. As such, the procedure was defined by an observable end point (i.e., a marked reduction in bubble release) rather than a fixed number of cycles, to account for the inherent variability in MC structure. As a broad reference, the number of applied cycles in this study ranged between 4 and 7.

After replacing the solution with fresh 0.2 M calcium acetate, the samples were left to soak for a total of either 5 or 24 h (hence, they are labeled as MC–CC05 and MC–CC24, respectively); this was done to control the degree of mineralization. During this period, the calcium acetate solution was stirred intermittently, and the samples were rotated periodically to ensure uniform solution infiltration. The samples were then removed from the solution and rotated at 30 min intervals under ambient conditions for 2 h to promote uniform, gravity-driven percolation of the residual solution and facilitate the evaporation of any excess solvent.

Subsequently, 0.4 M sodium bicarbonate solution was applied dropwise to all six surfaces of the calcium ion–impregnated cubic samples until they were visibly saturated. The samples were then left under ambient conditions for 10 min to allow equilibration before being fully immersed in the sodium bicarbonate solution for 5 (MC–CC05) or 24 h (MC–CC24), with intermittent rotation. Finally, the composites were air-dried for 2 h, and oven-dried at 90 °C for 48 h.

It should be noted that, consistent with the approach of Choi et al.,^23^ the samples were not rinsed between treatments or after mineralization. This methodological choice was made to prevent potential loss of loosely bound CaCO_3_ minerals.

### 2.4 Characterization

#### 2.4.1 Optical Characterization

Optical microscopy was carried out using a ZEISS Axio Zoom V16 microscope to qualitatively assess the presence and distribution of mineralization across the samples. Tests were conducted under ambient conditions, and images were acquired using reflected light.

#### 2.4.2 Compositional Analysis

Fourier transform infrared (FTIR) spectroscopy (PerkinElmer Spectrum 100 FT-IR Spectrometer) was used to qualitatively confirm calcium carbonate formation and provide insight into the likely calcium carbonate polymorphs.

#### 2.4.3 Quantification of Mass and Volume Change

Mineral deposition was quantified by calculating the percentage mass increase (wt %) of the MC samples following mineralization. It should be noted that these values represent gross mass changes, with contributions from the residual soluble salts in addition to the deposited carbonate as mineralized samples were not rinsed. Volumetric changes were similarly assessed as a percentage volume change (vol %), from the original state.

#### 2.4.4 Structural Analysis

Scanning electron microscopy (SEM) was used to examine the morphology of the mineralized mycelium composites, evaluate calcium carbonate characteristics (e.g., crystal size and distribution), and provide an indication of the possible polymorphic forms. The MC samples were mounted on aluminum stubs using conductive carbon tape and sputter-coated with a thin layer of gold/palladium (Au/Pd) alloy to minimize surface charging. Images were acquired using a Zeiss EVO 40 SEM operated at 10 kV with a secondary electron (SE) detector.

#### 2.4.5 Selective Area Electron Diffraction (SAED)

Selected area electron diffraction (SAED) was performed to confirm carbonate polymorphs, using a JEOL JEM-1400 transmission electron microscope (TEM). CaCO_3_ particles were extracted from the mineralized MCs by crushing bulk fragments and oxidizing in 30% hydrogen peroxide (H_2_O_2_, 30% w/w, lab grade, Merck, Germany) for 48 h, with fresh H_2_O_2_ added after 24 h. The resulting particles were repeatedly washed with deionized water until all residual matter was removed, then air-dried. It must be acknowledged that metastable CaCO_3_ polymorphs (e.g., vaterite) may be altered or dissolved during H_2_O_2_ treatment. Therefore, conclusions regarding CaCO_3_ polymorph distributions based solely on SAED should be interpreted with caution.

For SAED characterization, 5 mg of dried CaCO_3_ particles were dispersed in 60 mL deionized water and ultrasonicated in 30 s pulses for 10 min to avoid overheating. For each sample, a drop of dispersion was placed onto a carbon-coated copper grid and dried under ambient conditions. SAED patterns were obtained from selected regions and analyzed using the Crystallography Open Database (COD, https://www.crystallography.net/cod/) to identify crystal structures and corresponding lattice spacings.

#### 2.4.6 Thermal Characteristics

Thermogravimetric analysis (TGA) and derivative thermogravimetry (DTG) were performed to assess the thermal degradation stability, the onset of thermal degradation, and the peak degradation temperatures of the mineralized composites. The tests were carried out over a temperature range of 30–900 °C (under nitrogen flow), with a heating rate of 10 °C min^–1^, using an STA 449 F3 Jupiter simultaneous thermal analysis (STA) system.

#### 2.4.7 Characterization of Compressive Properties

Quasi-static compression tests were performed using a Shimadzu mechanical testing system equipped with a 1 kN load cell, operating in displacement-control with a crosshead displacement rate of 1 mm min^–1^. The tests were conducted until failure, which was defined as the maximum load the composites could sustain before the onset of visible cracking. Engineering compressive stress was calculated as the load divided by the original cross-sectional area of the sample and normalized by the respective densities of samples to account for inherent variations in this property. A noncontact video gauge extensometer was used to measure the deformation of the specimens in the loading direction, to enable calculation of strain independent of machine compliance. Strain was calculated as the percentage change in gauge length divided by the original gauge length of the sample.

#### 2.4.8 Water Contact Angle Measurements

Water contact angle (WCA) measurements were performed using a KRÜSS DSA100 drop shape analyzer to evaluate the surface wettability of the samples. The measurements were conducted on the dry surfaces of the as-prepared MCs, MC–CC05, and MC–CC24 samples. A 5 μL droplet of distilled water was used for each measurement.

## 3 Results and Discussion

### 3.1 Physical Characteristics

Figure 3 shows optical micrographs of the treated samples, confirming successful mineralization throughout the MC, as indicated by the presence of white dots on the surfaces and in the cores of the mineralized composites (Figure 3). The mineralization appears to be evenly distributed throughout the samples, both on the surface and within their cores. The specific polymorphs and particle sizes are identified in subsequent sections as it is not possible to do so with optical microscopy. It is noted that the white deposits observed in the micrographs may include residual sodium acetate, sodium bicarbonate, and other byproducts formed during the treatment, as the samples were not rinsed after the mineralization process.

**Figure 3:**
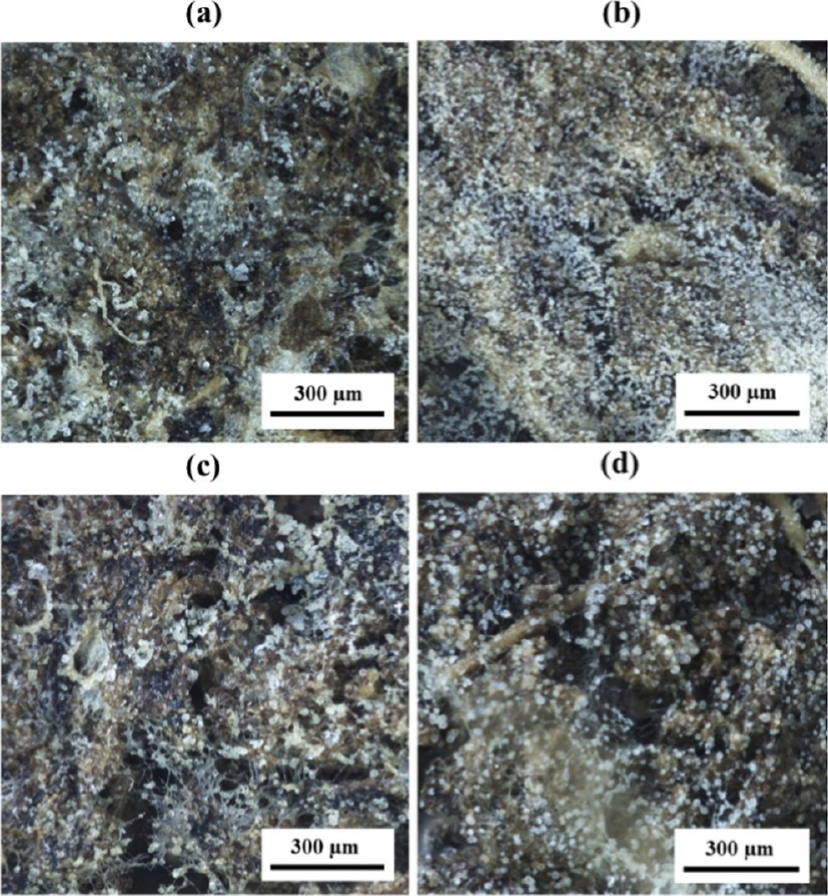
Representative optical micrographs showing the (a) surface and (b) core of MC–CC05 samples; (c) surface and (d) core of MC–CC24 samples. CaCO_3_ particles can be identified as white dots on the MCs.

Figure 4 presents an interval plot illustrating the mass gain (wt %) and volumetric expansion (vol %) of the treated samples. The MC–CC05 samples exhibited a mass gain of ∼0.9 wt %. The MC–CC24 samples however gained about ten times as much (i.e., ∼10.2 wt %) mass, which is likely due to the longer solution exposure period (and ion availability). It is important to note that, because the samples were not rinsed, the respective mass gains may also include contributions from sodium acetate (a byproduct) and residual sodium bicarbonate salts. MC–CC05 and MC–CC24 both show increase in their respective volumes after treatment. However, despite the significantly lower mass gain in the MC–CC05 samples, their volumetric expansion is comparable in magnitude to that of the MC–CC24 samples, i.e., ∼4.9 vol % for MC–CC05 and ∼6.0 vol % for MC–CC24.

**Figure 4:**
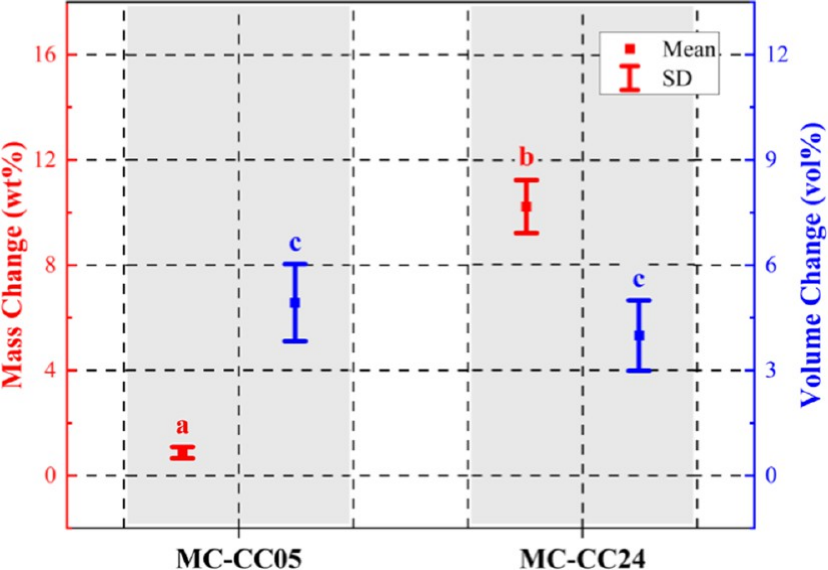
Means of mass (red) and volume changes (blue), with respective standard deviations (SD) shown as error bars, of MC–CC05 and MC–CC24 samples after mineralization treatment. Statistical differences among respective groups (i.e., mass change MC–CC05 vs MC–CC24; volume change MC–CC05 vs MC–CC24) were assessed using a one-way ANOVA test. A unique letter above the datum indicates a significant difference (*p* < 0.05, *n* = 8 samples per group).

In a preliminary water immersion test using deionized water, as-prepared MCs absorbed approximately 210 wt % moisture, consistent with literature reports of 40–560 wt % absorption under full water immersion,^43^ and exhibited a volumetric expansion of 11.1% after 5 h. Beyond this point, the absorption rate declined markedly, indicating that the material was approaching saturation. By 24 h, absorption reached ∼261 wt %, with a corresponding expansion of 13.5 vol %. This high rate of absorption can introduce osmotic and mechanical stresses in the MCs, leading to significant structural changes. These changes include pore formation or expansion due to, for example, hyphal damage or relaxation which limits the material’s ability to return to its original volume.^44,45^

Based on the observations from the water immersion test, it can be inferred that when the composites were immersed in the mineralization precursor solutions, the volumetric expansion of MC–CC05 was comparable to that of MC–CC24. However, the extended ionic availability for MC–CC24 samples allowed more extensive calcium carbonate nucleation and growth within the MC pore structure (Figure 5), resulting in an increased bulk density (Figure 6). In contrast, the substantial volumetric expansion of MC–CC05, despite its relatively small mass gain, indicates that the mineralization treatment likely induced significant pore dilation, microcracking, and other structural changes, with the lower bulk density reflecting the reduced CaCO_3_ content within the structure.

**Figure 5:**
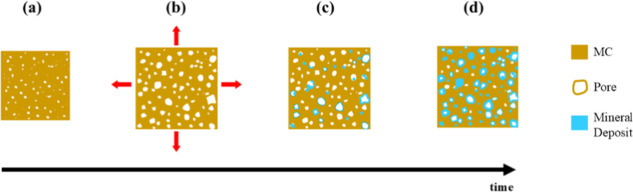
Schematic flowchart illustrating the temporal (time) evolution of mineralization: (a) as-prepared MC; (b) volumetric and pore expansion with solution infiltration; (c) mineral deposition after 5 h and (d) after 24 h.

**Figure 6:**
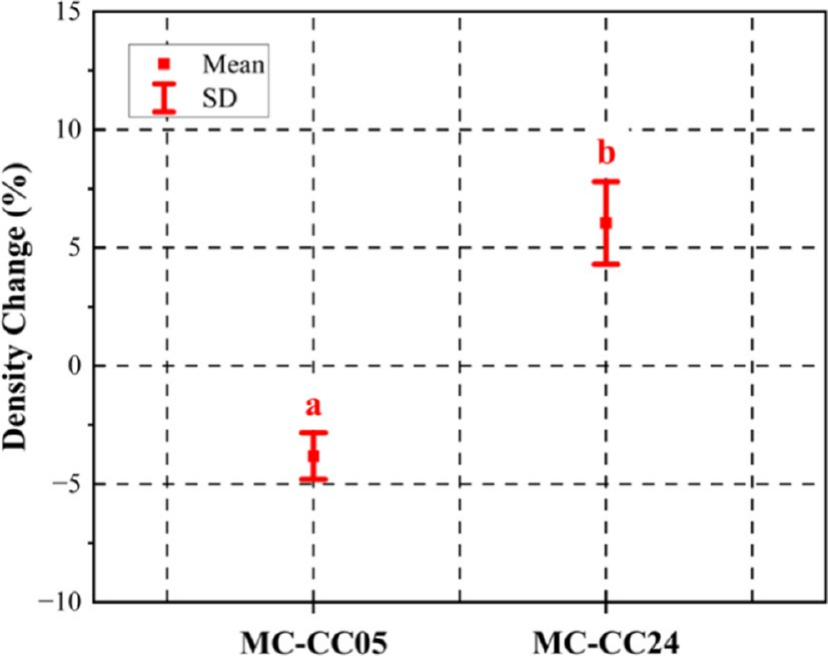
Change in density MC–CC05 and MC–CC24 samples after mineralization treatment. A unique letter above the datum indicates a significant difference (*p* < 0.05, *n* = 8).

### 3.2 Compositional Analysis

Figure 7 illustrates typical FTIR spectra of as-prepared mycelium composites before mineralization, along with those of raw coconut coir and lab-grown *P. eryngii* mycelium for comparison. The spectroscopic analysis was performed on both the surface and core regions of the composites to account for variability in mycelium content, which may result from differences in air availability during fungal growth.^46,47^ Consistent with previous literature,^7,11,48–51^ the composite spectra display characteristic bands corresponding to functional groups present in both the coconut coir and mycelium matrix. These include the broad band centered around ∼3300 cm^–1^ (O–H stretching vibrations), the band at ∼2922 cm^–1^ (aliphatic C–H stretching), and the polysaccharide fingerprint region spanning 1200–900 cm^–1^. While many of the bands in the latter region, primarily those attributed to C–O–C and C–O stretching vibrations, are shared by both the coir and the mycelium, the underlying polysaccharides differ: in the coir, they are attributed to cellulose and hemicellulose, whereas in the mycelium matrix, they are associated with chitin and glucan, the main structural polysaccharides in fungal organisms.^52^

**Figure 7:**
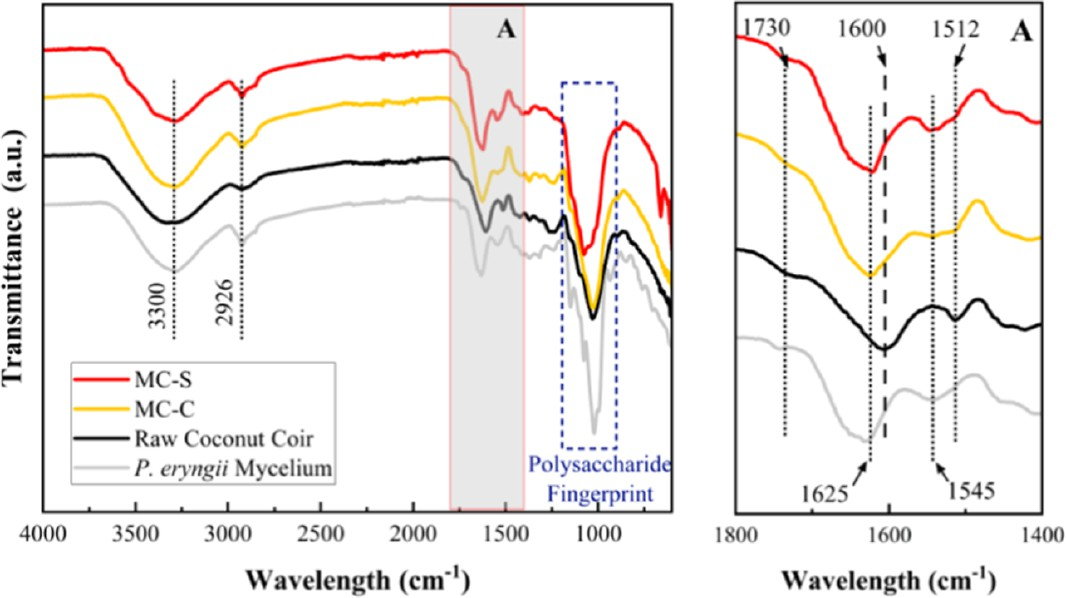
Representative FTIR spectra of the surface (MC-S) and core (MC-C) of as-prepared mycelium composites (MCs), compared with raw coconut coir and lab-grown *P. eryngii* mycelium, indicating the ‘Polysaccharide Fingerprint’ region (purple dashed box) and the presence of the 3300 and 2926 cm^–1^ bands; A = zoomed-in view of the protein fingerprint region (gray box), showing the presence of the 1730, 1625, 1600, 1545, 1512 cm^–1^ bands.

Comparison of the characteristic bands in the protein fingerprint region (gray box in Figure 7), spanning from ∼1700 to ∼1500 cm^–1^, enables differentiation between the coconut coir and mycelium phases, owing to their distinct spectral features. For instance, the C=O stretching vibration at ∼1625 cm^–1^, attributed to amide I groups, along with the N–H bending and C–N stretching of amide II at ∼1545 cm^–1^, confirms the presence of mycelium in both the core and surface regions of the composites.^53^ On the other hand, the C=O stretching of carbonyl groups for hemicelluloses at ∼1730 cm^–1^ and the C=C aromatic skeletal vibrations from lignin located at ∼1512 cm^–1^ are associated with the lignocellulosic coir.^54^ The lower intensity of the latter band may be due to an overlap with the amide II band (∼1545 cm^–1^) in mycelium.

This analysis confirms the presence of both proteins and carbohydrates, thus, making the MC scaffold also suitable for mineralization as these compounds provide functional groups for mineral nucleation.^30,31^ Figure 8 presents the FTIR spectra of the mineralized MC samples, revealing characteristic bands for calcium carbonate, further confirming the successful mineralization. The spectroscopic analysis was performed on both the surface and core regions of the MC samples to account for compositional variability. The bands associated with calcium carbonate include the band located at ∼1412 cm^–1^, attributed to the asymmetric stretching of carbonate ions; the band located at ∼873 cm^–1^, corresponding to the out-of-plane bending mode of carbonate ions; and the low-intensity band located at ∼713 cm^–1^ associated with in-plane bending of carbonate ions.^55–59^

**Figure 8:**
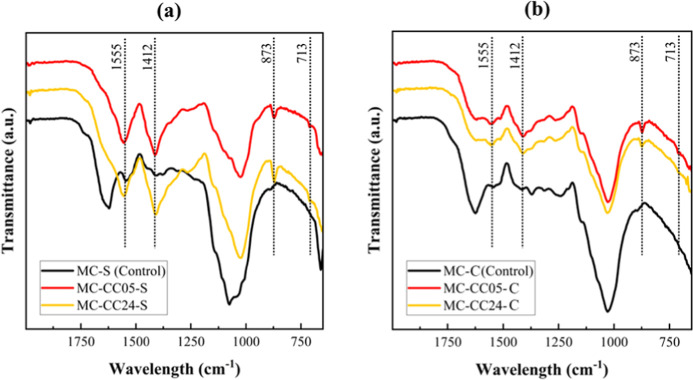
Representative FTIR spectra of (a) surface (MC–CC05–S and MC–CC24–S) and (b) core (MC–CC05–C and MC–CC24–C) of mineralized MCs compared with their respective controls (MC-S and MC-C). Dotted lines indicate the 1555, 1412, 873, and 713 cm^–1^ bands associated with calcium carbonate.

These spectral features, are clearly visible for the surface spectra of the samples (Figure 8a), and are typically associated with the calcite polymorph.^60,61^ Notably, these bands exhibit a lower intensity for the spectra obtained from core material (Figure 8b), where the complex network structure may have restricted ion mobility, resulting in decreased mineral deposition.

The high-intensity band located at ∼1555 cm^–1^ likely corresponds to the asymmetric stretching vibration of carboxylate groups coordinated with calcium ions in a bridging mode.^62^ This suggests the occurrence of mineral chemisorption of calcium carbonate onto the surfaces of the mycelium composite constituents.^63^ It should be noted that the bands located at ∼1555 and ∼1412 cm^–1^ may not exclusively indicate calcium carbonate, as these bands can also arise from proteins present in the mycelium or residual sodium acetate, where they correspond to the asymmetric and symmetric stretching vibrations of carboxylate (COO^–^) groups, respectively.^64^ This spectral overlap may contribute to the elevated band intensities observed for the surface material.

### 3.3 Polymorphic Characterization

The SEM images in Figure 9 and SAED patterns in Figure 10 provide further insight into the carbonate polymorphs and degree of mineralization. Figure 9a shows that the surface of MC–CC05 contains a relatively higher concentration of calcium carbonate particles compared to the core region (Figure 9b). This contrast becomes more pronounced in MC–CC24, where the surface (Figure 9c) exhibits an even concentration relative to the core (Figure 9d). Except for the MC–CC05 cores, all other regions (i.e., MC–CC05 surface, MC–CC24 surface, and MC–CC24 core) display polycrystalline structures of vaterite and calcite. Vaterite appears as near-spherical (Figure 9e) crystals, whereas calcite is observed as multifaceted crystals (Figure 9f) or aggregates (Figure 9g).^65,66^ Given that polymorphic transformation is time-dependent, the presence of larger calcite aggregates in MC–CC24 is expected (Figure 9c), as prolonged exposure likely supplied ample calcium and carbonate ions for crystal growth.

**Figure 9:**
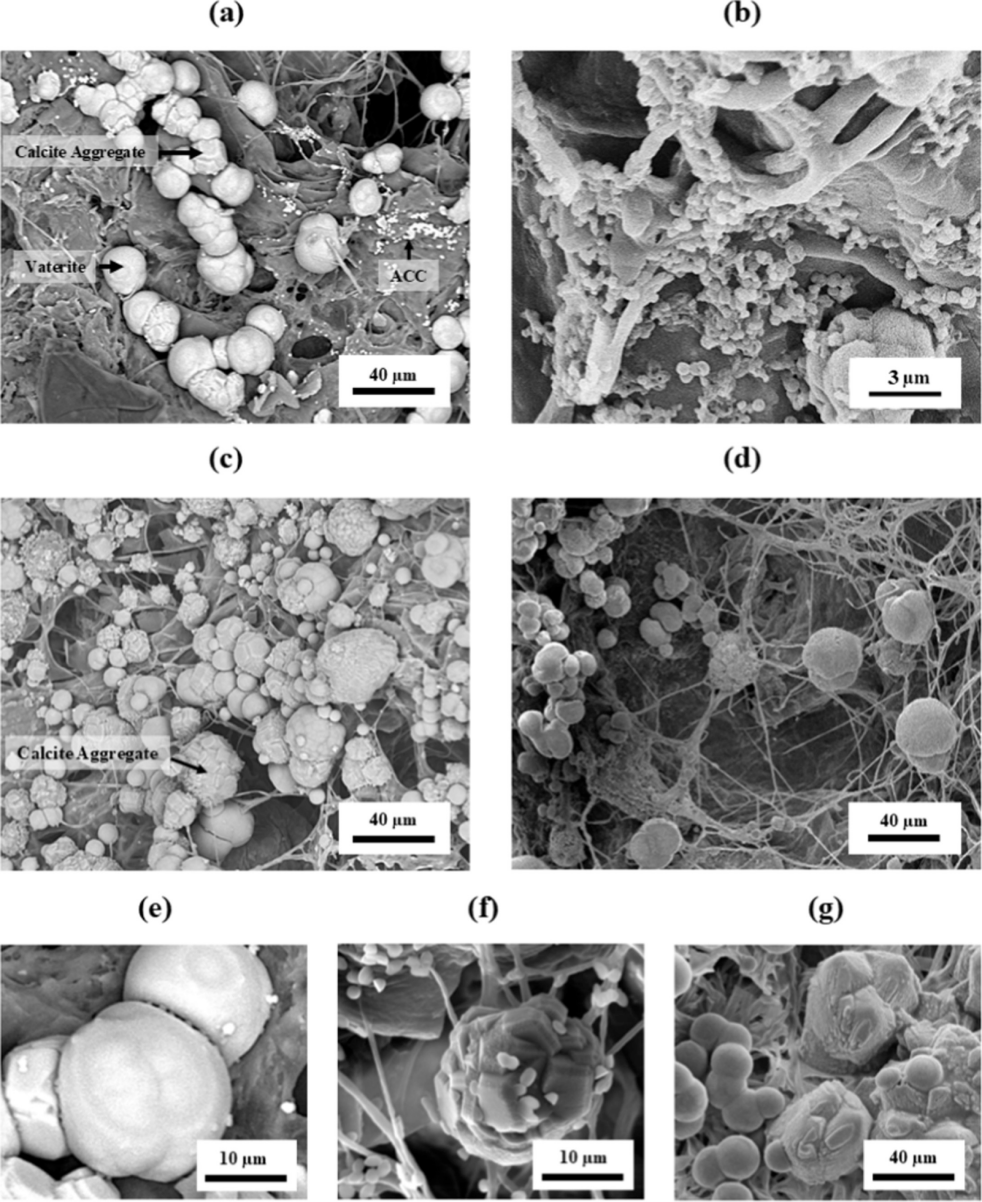
(a,b) Representative SEM images of MC–CC05: (a) surface showing polycrystalline mineralization and (b) core showing amorphous mineralization. (c,d) SEM of and MC–CC24: (c) surface and (d) core, both exhibiting polycrystalline mineral formation. Black arrows indicate different types of CaCO_3_ particles identified in the images. Representative SEM images of (e) vaterite, (f) rhombohedral calcite, and (g) multifaceted calcite aggregates identified on mineralized MCs.

**Figure 10:**
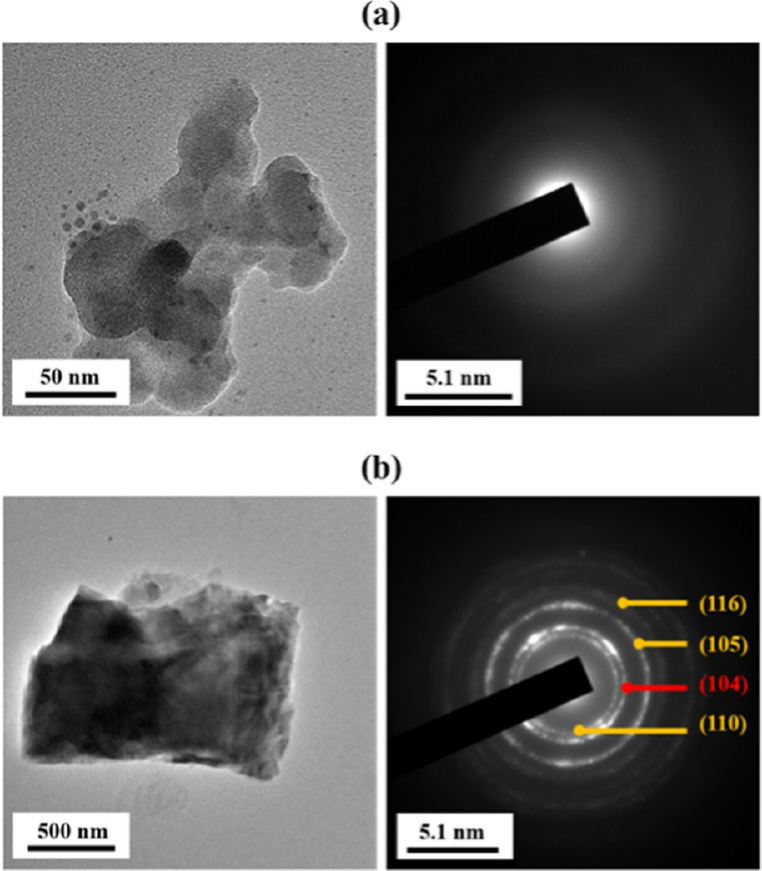
Representative TEM images (left) and respective SAED patterns (right) of (a) amorphous calcium carbonate (ACC) and (b) crystalline calcium carbonate. Miller indices in the SAED images correspond to lattice planes of vaterite (yellow: (116), (105), (110)) and calcite (red: (104)) polymorphs.

On the other hand, the SEM image of the MC–CC05 core (Figure 9b) shows sparsely distributed nanoscale particles, i.e., amorphous calcium carbonate (ACC).^67^ This is due to the shorter treatment time for MC–CC05 samples and limited ion supply, resulting in a lower degree of mineralization.

Figure 10 shows representative TEM images (left) and corresponding SAED patterns (right) of an amorphous calcium carbonate particle and a polycrystalline particle extracted from the mineralized MCs. From the TEM images, the amorphous particle appears as (Figure 10a) a cluster of globular nanosized particles, similar to the particles observed in the SEM.^68^ In constrast, polycrystalline particles (Figure 10b) exhibit multifaceted morphologies.^69^ The SAED patterns further support the morphological observations from the SEM and TEM analyses. A broad, diffuse halo (Figure 10a) was detected exclusively in the core of MC–CC05. In contrast, distinct Bragg diffraction rings (Figure 10b) were observed for CaCO_3_ extracted from the surface and core of MC–CC24 samples as well as the surface of MC–CC05 samples. These rings correspond to the calcite and vaterite polymorphs.^70^ This is confirmed by the identified lattice planes, confirming the coexistence of multiple crystalline polymorphs.

### 3.4 Thermal Analysis

The TGA and DTG curves in Figure 11 demonstrate the thermal stability and decomposition profiles of the samples under inert conditions (N_2_). As-prepared MCs undergo five distinct stages of thermal degradation, namely.

1. Evaporation of moisture and the release of volatile extractives (30–125 °C).^71^
2. Decomposition of polysaccharide side chains (e.g., α-glucans and branched glucomannan) and glycoproteins in the mycelial cell walls (120–175 °C).^72^
3. Degradation and volatilization of β-glucans in the mycelial cell walls,^73^ as well as the breakdown of hemicelluloses in the coconut coir, occurring between 125 and 300 °C (shoulder peak).^71^
4. Cellulose degradation at 300–400 °C^74^
5. Lignin degradation spanning from 400 to 800 °C.^74^

**Figure 11:**
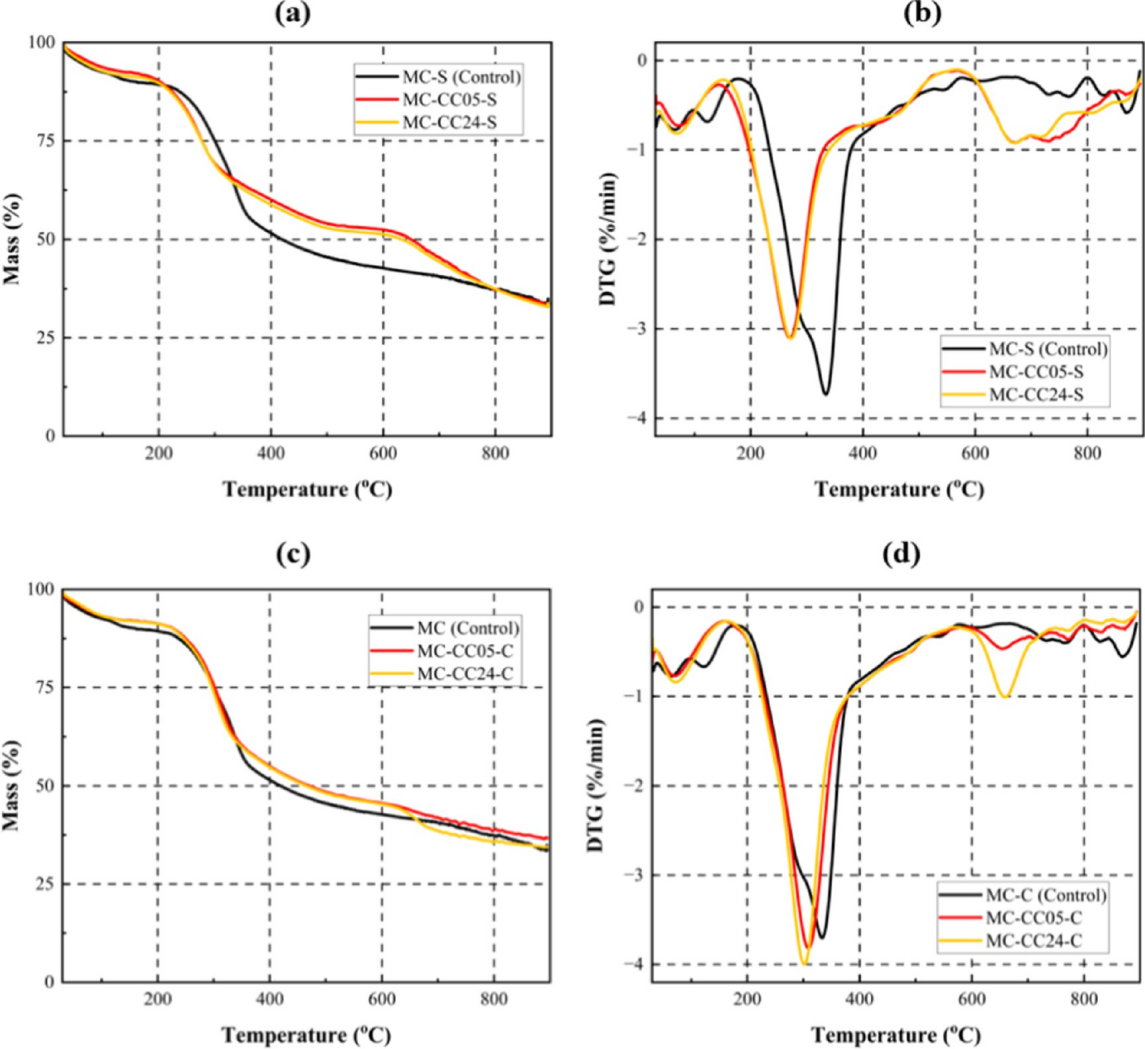
(a) TGA and (b) DTG curves of the surface regions, and (c) TGA and (d) DTG curves of the core regions of mineralized samples compared to as-prepared mycelium composites.

However, as seen in Figure 11 the thermal degradation behavior of the mineralized samples deviates from this, exhibiting the following key differences.

1. Disappearance of the 120–175 °C degradation stage.
2. Shift and merging of the degradation peaks of hemicellulose and cellulose, originally spanning from 190 to 390 °C with an onset degradation temperature of ∼260 °C, into a single peak with an earlier onset of degradation, particularly at the surface of mineralized samples (∼208 °C).
3. Emergence of broad peaks between 600 and 900 °C (∼600 °C onset degradation), corresponding to the thermal decomposition of calcium carbonate into CaO and CO_2_.^60^ This peak is especially prominent in the DTG curves of the surface regions, where carbonate content is higher.

The absence of the polysaccharide/glycoprotein peak, coupled with the altered cellulose degradation profile, suggests that these components were partially degraded or structurally modified during the mineralization treatment, resulting in reduced thermal stability. Although consistent with Choi et al.,^23^ who noted similar behavior in mineralized wood, this finding was unexpected as CaCO_3_ is commonly associated with increased thermal performance in natural composites.^75–77^

While the literature specifically addressing the causes of reduced thermal stability in mineralized materials is limited, the work by Zhou et al.,^78^ albeit in a different context (i.e., torrefaction), offers valuable insights into this phenomenon. In their study on the effects of alkaline earth metal salts during cellulose torrefaction, they observed that calcium salts catalyze cellulose degradation by forming carboxylate complexes through ionic bridging with carboxylic groups on cellulose. This interaction disrupts the native hydrogen-bonded network, reducing crystallinity and thereby lowering the activation energy for degradation and overall thermal stability.^78,79^ For context, torrefaction is a low-temperature thermal pretreatment in which biomass is heated at 200–300 °C under an inert atmosphere.^80^ Given that ligninolytic activity by the mycelium species exposes cellulose in the lignocellulosic substrate,^81,82^ and considering that the TGA/DTG analysis was conducted under inert conditions, parallels can be drawn between the early stages of the thermal degradation profile (low temperature region) and the torrefaction process. Thus, the decline in thermal stability may be attributed to the catalytic effect of calcium carbonate on the degradation of exposed cellulose and hemicellulose. Future work should focus on a more comprehensive investigations to better understand this phenomenon, particularly within the specific context explored in this study.

### 3.5 Compressive Properties

Figure 12 presents compressive stress-strain curves (with first-order derivatives) and interval plots of normalized stress and strain at failure for MC–CC05 and MC–CC24 samples, respectively. The observed variability between the controls for MC–CC05 and MC–CC24 arises from inherent batch-to-batch differences, which are typical of MCs and other biobased composites.^8^ For each sample group (MC–CC05 and MC–CC24) and their respective controls, eight replicate samples were prepared from the same culture batch (i.e., all MC–CC05 samples and their controls were obtained from one culture, and all MC–CC24 samples and their controls were obtained from a second culture batch). This approach ensured consistency within groups, although it may limit the generalizability of the results across different batches.

**Figure 12:**
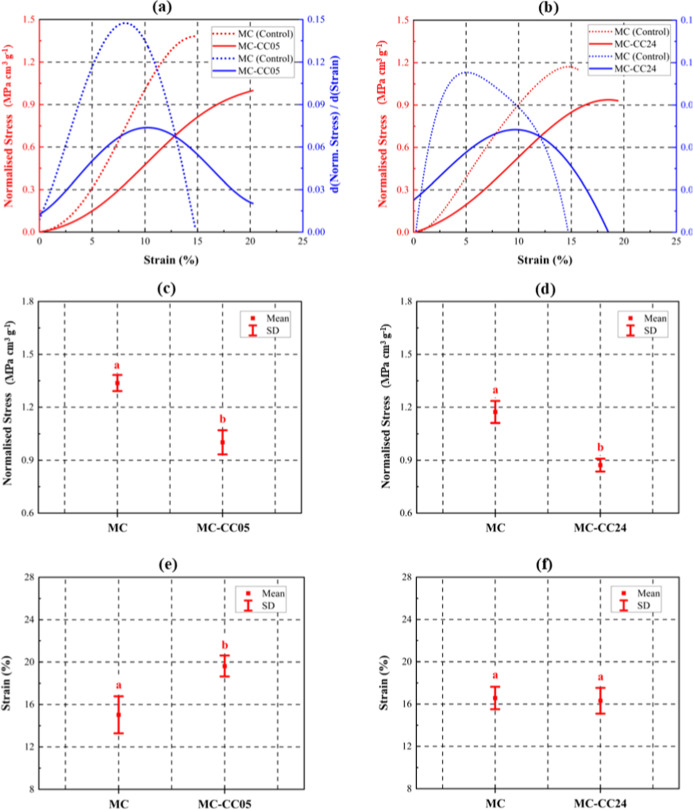
Representative (a,b) stress–strain curves, (c,d) interval plot of compression stress, and (e,f) interval plot of strain as failure of MC–CC05 and MC–CC24 samples compared to their respective as-prepared control MC samples. Statistical differences among groups were assessed using a one-way ANOVA test. A unique letter above the datum indicates a significant difference (*p* < 0.05, *n* = 8 samples per group).

The stress–strain curves indicate that both MC–CC05 and MC–CC24 samples exhibit a nonlinear deformation behavior similar to that of the as-prepared MC control samples. This behavior is characterized by an initial increase in stress due to nonlinear stiffening under load, followed by a sharp drop corresponding to structural failure. In a previous study, the initial stress increase (below 8% strain) prior to strain hardening was attributed to linear elastic deformation.^83^ However, based on the findings of this study, a linear elastic response is considered unlikely, even in the low strain region, as it would result in a constant first derivative.^84^

Mineralization led to a reduction in compressive strength, with MC–CC05 and MC–CC24 exhibiting decreases of approximately 24.2% and 19.8%, respectively, compared to untreated control samples. MC–CC05 demonstrated a notable increase in strain at failure (∼30.6%), while MC–CC24 showed a slight reduction (∼1.5%). These findings align with Choi et al.,^23^ who attributed the reduced strength to a low CaCO_3_ volume fraction stemming from (1) restricted infiltration of calcium chloride and sodium carbonate solutions due to the narrow and tortuous pore structure and increased capillary resistance with repeated treatment cycles; (2) insufficient organic nucleation sites in wood to guide mineral growth, and (3) rapid in situ precipitation of CaCO_3_ near the surface before deep infiltration, causing uneven mineral distribution.

The observed reduction in strength may also be attributed to the significant volumetric expansion (Table 1) upon exposure to the precursor solvents during mineralization. Given the weak interfacial bonding between mycelial hyphae and substrate particles, along with the inherently fragile nature of the hyphae, the capillary and mechanical stresses induced by the substantial uptake of precursor solvents likely caused internal delamination and microstructural damage. This was not observed in mineralized wood, for example, likely owing to the inherent stiffness and strength of natural wood derived from its composition and hierarchical microstructure,^85^ which would confer better dimensional stability and reversible swelling under certain conditions (e.g. type of wood, solvent composition, etc.).^86^ Wood has a multilayered cell wall architecture consisting of highly crystalline cellulose microfibrils embedded in a lignin–hemicellulose matrix, tightly bound through hydrogen bonding and van der Waals forces.^85^ These interactions form a dense, mechanically robust network that enhances stiffness and structural strength, thus, reducing swelling-induced damage.

**Table 1:** Summary of Percentage Changes (Reported as Mean % ± Percentage Standard Error of Mean) in Physical and Mechanical Properties of MC–CC05 and MC–CC24 Samples as Compared to Their Respective Control Samples

sample ID	change in absolute stress (%)	change in strain (%)	density change (%)	mineral mass gain (wt %)	volumetric expansion (vol %)
MC–CC05	–27.2 ± 4.1	+30.6 ± 14.2	–1.6 ± 2.4	+0.9 ± 0.1	+4.9 ± 0.7
MC–CC24	–19.8 ± 4.8	–1.5 ± 6.7	+8.2 ± 2.1	+10.2 ± 0.6	+4.0 ± 0.6

### 3.6 Water Contact Angle Analysis

Figure 13 presents the surface contact angle images for MC–CC05 and MC–CC24 compared to untreated MC control samples. In agreement with the literature, MCs samples exhibit high surface hydrophobicity,^87,88^ with a contact angle of ∼130°, attributed to the high protein (particularly mannoproteins and hydrophobins)^89^ and lipid contents^90^ in the cell wall of mycelia.^91^

**Figure 13:**
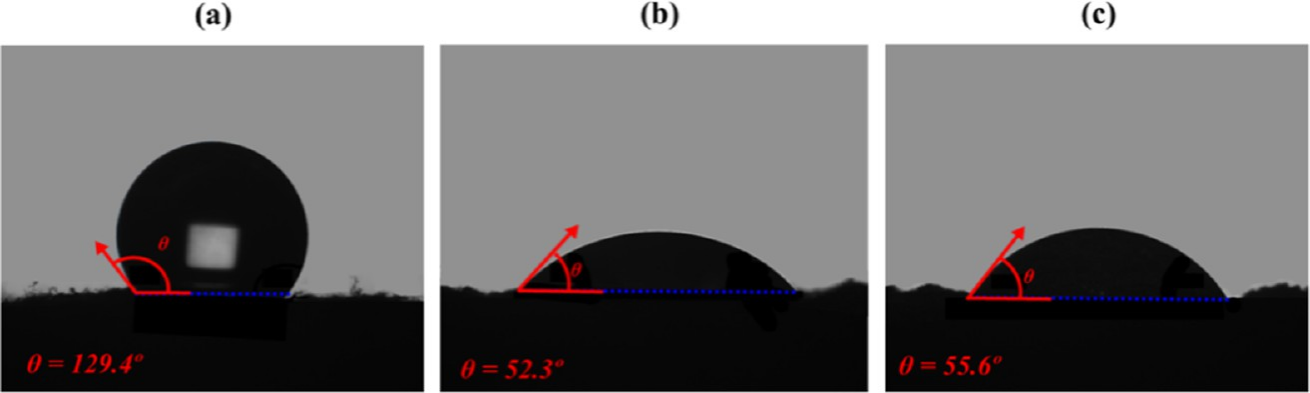
Representative WCA images, showing the respective contact angles (θ) of (a) as-prepared MCs (θ = 129.4°), (b) MC–CC05 (θ = 52.3°), and (c) MC–CC24 (θ = 55.6°) samples.

However, the incorporation of CaCO_3_ increased surface hydrophilicity, with MC–CC05 and MC–CC24 exhibiting more than a 50% reduction in water contact angle compared to untreated samples. This was expected as CaCO_3_ is inherently hydrophilic.^92^ Moreover, structural changes (e.g., volumetric expansion and contraction) during mineralization and drying may have damaged the native hydrophobic mycelial layer, thus exposing the underlying hydrophilic lignocellulosic coconut coir and further increasing surface wettability. It should be noted that the relatively higher contact angle of MC–CC24 compared to MC–CC05 is likely due to localized surface variations and inherent sample-to-sample variability, which is common with natural materials.

The increased hydrophilicity presents several drawbacks.^93,94^ First, the marked drop in moisture resistance reflects a significant decline in a key functional property of MCs, reducing their suitability for applications such as packaging and insulation. Furthermore, elevated water uptake can promote microbial colonisation and degradation, accompanied by an unpleasant odor, posing health and hygiene concerns. It can also cause hydrolytic degradation and plasticization of the organic components, as well as induce environmental microcracking due to cyclic swelling and shrinking. Ultimately, these factors could compromise the mechanical integrity of the composite, and so this is something that should be addressed if these composite materials are truly going to be used in practical applications, such as in construction.

## 4 Limitations and Future Considerations

This study was designed to explore whether a calcium carbonate (CaCO_3_) mineralization method commonly applied to wood could be transferred to mycelium composites (MCs). While the work provides valuable insights, several limitations inherent to the methodology and the material system must be acknowledged.

1. Methodological transfer from wood to MCs: The mineralization protocol was adapted from wood without modification for the unique microstructure and chemistry of MCs. Unlike wood, MCs rely on weak physical and secondary bonding between hyphae and substrate particles, making them sensitive to solvent exposure. Even brief immersion in water or precursor solutions can induce swelling or structural disruption, likely contributing to the observed reduction in compressive strength. Future strategies should consider MC-specific conditions, including lower precursor concentrations, stepwise or pulsed mineralization, slower nucleation regimes, or alternative methods that avoid bulk solvent penetration.
2. Limited experimental scope and controls: Only two mineralization durations (5 and 24 h) and a single precursor concentration were tested. Additional controls—such as solvent-only treatments, precursor-only exposures, and rinsed versus nonrinsed samples—would help isolate the effects of mineralization from solvent-induced structural changes. Including these controls in future studies would provide a clearer mechanistic understanding of the observed property changes.
3. Species- and substrate-specificity: This study focused exclusively on *P. eryngii* grown on a coconut-coir substrate. Since MC properties are strongly influenced by fungal species and substrate chemistry, the conclusions drawn in this mineralization study must also be specifically linked to the chosen mycelium-substrate system. Exploring different fungal species and substrates may reveal systems that respond more favorably to mineralization, potentially producing improved composite properties.
4. Biochemical residues and substrate chemistry: Partial delignification by fungal species exposes hydroxy (−OH) groups on the cellulose and hemicellulose components, which can theoretically serve as nucleation sites for CaCO_3_. However, enzymatic degradation also produces a complex mixture of residues—partially degraded polysaccharides, lignin fragments, organic acids, and proteins—which, combined with unwashed mineralization byproducts (e.g., sodium acetate, bicarbonate salts), creates a heterogeneous chemical environment. This complexity may interfere with nucleation, mineral growth, and polymorph stabilization, limiting the efficiency of mineral deposition, potentially attributable to the observations of this study.
5. Polymorph composition and particle size: Although FTIR and SAED confirmed the presence of calcite and vaterite, their relative proportions, particle size distribution, and impact on MC properties were not evaluated. SEM images indicate relatively large, discontinuous mineral deposits, which may act as stress concentrators and compromise the organic matrix, reducing mechanical strength and thermal stability. Future work should quantify these relationships and investigate methods to produce finer, more uniform deposits to enhance composite performance.
6. Thermal behavior: The unexpected thermal behavior was attributed to a potential catalytic effect of CaCO_3_ on the degradation of cellulose exposed by the ligninolytic activity of mycelium, a conclusion that was drawn from the previous literature on wood. However, in MCs, the chemical complexity introduced by residual metabolites and unwashed salts may also influence thermal stability. A more detailed thermal and chemical analysis is required to clarify the mechanisms involved.
7. Functional performance beyond mechanics: This study did not assess application-specific properties such as fire resistance or durability. Even though mineralization did not enhance mechanical properties, it may offer other benefits, which should be explored in future work.

## 5 Conclusions

CaCO_3_ was successfully formed on both the surface and within the MCs using a combination of vacuum, gravity, and mechanical assistance, with calcium acetate and sodium bicarbonate as precursors. This process yielded approximately 1 and 10 wt % of CaCO_3_ (and other residues) after 5 h and 24 h treatments, respectively. While the main aim of this study was to enhance the compressive strength by mimicking natural biomineralization processes, the findings of this study reveal that the approach introduces several significant drawbacks. First, the compressive strength of the composites was significantly reduced, likely due to inhomogeneous mineral distribution, insufficient densification of the material, and internal structural damage induced by solvent absorption, volumetric expansion, and drying-related stresses leading to mechanical breakdown of the structure. Interestingly, even the thermal stability of mineralized MCs was compromised, suggesting that the calcium salts may have a catalytic effect on the thermal degradation of the organic matrix. Furthermore, as expected, hydrophilicity increased, with mineralized MCs showing a >50% reduction in water contact angle; this would subsequently compromise their dimensional stability, durability, and resistance to biological degradation. While this may be viewed as a negative effect, there are some interesting parallels with other porous solids e.g., wood, and the need to report these data to assist with future work is demonstrated in light of these.

These findings highlight the complexity of functionalizing mycelium composites through mineralization and provide valuable insights into potential areas for improvement. Despite current limitations, biomineralization of mycelium composites remains a promising approach, provided the manufacturing protocol is optimized for their structural and compositional features and processing-induced damage is minimized. Future efforts should focus on controlling deposition kinetics, enhancing precursor infiltration, and mitigating solvent-induced structural damage to improve the mechanical performance of mineralized MCs
